# Severity of comorbid conditions and early-stage breast cancer therapy: linked SEER-medicare data from 1993 to 2005

**DOI:** 10.1002/cam4.66

**Published:** 2013-06-24

**Authors:** Shagufta Yasmeen, Rowan T Chlebowski, Guibo Xing, Cyllene R Morris, Patrick S Romano

**Affiliations:** 1 University of CaliforniaDavis, California; 2 Harbor-UCLA Research and Education InstituteTorrance, California; 3 California Cancer RegistrySacramento, California

**Keywords:** Comorbidities, early stage breast cancer, racial disparities, treatment

## Abstract

Comorbidity burden has been suggested as influencing early-stage breast cancer therapy but previous studies have not considered the severity of these comorbidities. Therefore, we examined the influence of comorbidity severity by age and race/ethnicity on early-stage breast cancer treatment over time. We used linked Surveillance, Epidemiology, and End Results (SEER)-Medicare data to determine whether comorbidity severity influences receipt of definitive and preferred early-stage breast cancer treatment and explains racial/ethnic and age disparities in receiving such therapy. Definitive surgical therapy was defined as any primary surgery other than breast conserving surgery (BCS) without radiation therapy (RT). Preferred surgical therapy was defined as BCS plus RT. Comorbidities were defined as either “unstable” (life threatening or difficult to control) or “stable” (less serious but with potential to influence daily activity). Surgical treatment trends from 1993 to 2005 were analyzed in regression models adjusting for comorbidity burden, age, and race/ethnicity in 93,596 elderly female Medicare beneficiaries with stage 1–2 invasive breast cancer. Receipt of BCS alone (compared with any definitive surgical therapy) was independently associated with neighborhood socioeconomic status, unmarried status (OR [odds ratio] 1.18, 95% CI: 1.12–1.23), tumor size (OR 0.78, 95% CI: 0.69–0.87 for tumors ≥4 cm vs. <2 cm), tumor grade (OR = 0.89, 0.88, and 0.81 for grades 2–4 vs. 1, respectively), stable comorbidities (OR = 0.76, 0.71, and 0.72 for 1, 2, and 3 vs. 0 stable comorbidities, respectively), and unstable comorbidities (OR 1.20, 95% CI: 1.14–1.28). Black women were 4–5% more likely to receive suboptimal therapy (BCS alone), even after adjusting for all available patient, tumor, and regional characteristics. Black race/ethnicity was associated with higher probability of receiving suboptimal treatment, independent of comorbidities, although we do not know whether this effect was due to clinicians' failure to offer RT or patients' failure to accept it.

Comorbidities, especially unstable comorbidities, adversely influence receipt of definitive and preferred early stage breast cancer therapy.

## Introduction

Breast conserving surgery plus radiation therapy (BCS + RT), or mastectomy (MST) (plus RT for women with large tumors or >3 lymph node involvement), constitutes definitive surgical therapy for women with early-stage (I, II) breast cancer 1–4. In this setting, BCS + RT is the preferred treatment over MST due to its less invasive nature and associated breast preservation 1–5.

Despite the effectiveness of BCS + RT 4–6, older Black women are less likely than older White women to receive RT following BCS even after adjusting for socioeconomic status, comorbidities, and tumor characteristics 7,8. Racial/ethnic disparities in breast cancer therapy with associated shortened survival have, in part, been attributed to a higher comorbidity burden among older and Black women 10. However, the data supporting this association have limitations as the same weight is often given to all comorbidities, despite their differing impact on life function and expectancy. Therefore, our study objective was to determine whether the presence and severity of comorbid conditions account for differences in early-stage breast cancer treatment across racial/ethnic groups and across age categories. We hypothesized that better adjustment for comorbidity, and accounting for interactions involving comorbidity, would diminish racial/ethnic and other demographic differences in use of definitive and preferred therapy among women with early-stage breast cancer at diagnosis. Persistence of differences in the use of definitive and preferred therapy, despite these methodologic enhancements, would highlight the need for continuing attention to disparities in oncologic care.

## MATERIALS AND METHODS

### Data sources and sample selection

We used linked Surveillance, Epidemiology, and End Results (SEER)-Medicare data from 1993 to 2005 to estimate the likelihood of receiving definitive and preferred early-stage breast cancer therapy in relation to the presence and severity of comorbid conditions using an updated approach to classify comorbidities based on severity 11.

Our analyses incorporated data from the linked SEER program and Medicare Part A Hospitalization and Part B Physician, Supplier, and Outpatient Facility Claims. The SEER program includes population-based tumor registries that cover approximately 14% of the U.S. population 12 and capture data on all incident cancers (except nonmelanoma skin) 12–13 with information on tumor location, stage, hormone receptor status, demographic characteristics and surgery and RT received 14.

The Medicare program covers more than 97% of Americans aged 65 years or more. The Medicare claims data used in this study included: (1) the Medicare Provider Analysis and Review (MEDPAR) file; (2) the Hospital Outpatient Standard Analytic File; and (3) 4,15 the 100% Physician/Supplier file 1. All of these files include service dates and diagnostic and procedure codes. The MEDPAR file contains a summarized record of each hospital inpatient stay, with up to 10 diagnoses and procedures coded using the International Classification of Diseases, 9th Revision, Clinical Modification (ICD-9-CM 12–18. These codes can be used to capture information on surgical procedures, radiation treatment, and chemotherapy administration. The hospital outpatient file and the physician claims file use Healthcare Common Procedure Coding System (HCPCS) codes, which incorporate the American Medical Association Common Procedure Terminology codes (CPT-4) 10, with additional codes used exclusively by CMS.

The SEER registry reported 118,742 women with primary invasive breast cancer between 1 January 1993 and 31 December 2005 11. Information on surgical procedures and RT was available for 107,536. Of those, 93,596 (87%) women were diagnosed with localized stage cancer (stage 1, 11a, and 11b) according to the American Joint Committee on Cancer (AJCC) staging system 14 after excluding 11,206 (9.4%) women for unknown information on stage at diagnosis ( 1).

**Table:** Receipt of major types of surgical treatment among female medicare beneficiaries diagnosed with early-stage (stages 1, 2a, 2b) breast cancer from 1993 to 2005 (*N* = 93,596)

Variables	All treatments			BCS alone^5^ *N* = 13,144					BCS + RT^6^ *N* = 39,691							MST ± RT^7^ *N* = 38,631									*P* value
	*N* (%)			*N* (% receiving)					*N* (% receiving)							*N* (% receiving)									
Year									
1993–1997	31,302	(33)	4211	(13)	10,859	(35)	14,950	(48)	<0.001
1998–2002	39,416	(42)	5466	(14)	17,465	(44)	16,045	(41)	
2003–2005	22,878	(24)	3467	(15)	11,367	(50)	7636	(33)	
Age in years									
67–69	15,490	(17)	1171	(8)	8004	(52)	6019	(39)	<0.001
70–74	26,521	(28)	2096	(8)	13,222	(50)	10,697	(40)	
75–79	24,073	(26)	2661	(11)	10,848	(45)	10,110	(42)	
80–84	16,333	(17)	3074	(19)	5687	(35)	7198	(44)	
85–89	7975	(9)	2608	(33)	1647	(21)	3438	(43)	
90+	3204	(3)	1534	(48)	283	(9)	1169	(36)	
Race/ethnicity									
White	81,165	(87)	11,346	(14)	34,884	(43)	33,161	(41)	<0.001
Black	5083	(5)	888	(17)	1806	(36)	2208	(43)	
Hispanic	3800	(4)	485	(13)	1595	(42)	1610	(42)	
Asian^1^	3020	(3)	296	(10)	1212	(40)	1471	(49)	
Other^2^	528	(1)	129	(24)	194	(37)	181	(34)	
Area of residency									
Big metro	52,907	(57)	7755	(15)	23,884	(45)	19,968	(38)	<0.001
Metro	27,782	(30)	3622	(13)	11,627	(42)	11,830	(43)	
Urban	5508	(6)	752	(14)	2184	(40)	2517	(46)	
Less urban/rural	7399	(8)	1015	(14)	1996	(27)	4316	(58)	
Patients living in a census tract where at least 25% of residents age ≥25 years do not have high school diploma									
Missing	1368	(1)	248	(18)	530	(39)	542	(40)	<0.001
Yes	20,545	(22)	3063	(15)	7074	(34)	9785	(48)	
No	71,683	(77)	9833	(14)	32,087	(45)	28,304	(39)	
Patients living in a census tract where at least 25% of households are below poverty level									
Missing	1368	(1)	248	(18)	530	(39)	542	(40)	<0.001
Yes	22,229	(24)	3344	(15)	7823	(35)	10,441	(47)	
No	69,999	(75)	9552	(14)	31,338	(45)	27,648	(40)	
Marital status									
Unmarried	51,280	(55)	8697	(17)	19,249	(38)	22,053	(43)	<0.001
Married	39,409	(42)	3823	(10)	19,373	(49)	15,495	(39)	
Unknown	2907	(3)	624	(21)	1069	(37)	1083	(37)	
AJCC stage									
1	56,293	(60)	8855	(16)	28,683	(51)	17,701	(31)	
2	37,303	(40)	4289	(12)	11,008	(30)	20,930	(56)	
Tumor size									
0 to <2 cm	49,241	(53)	6849	(14)	24,707	(50)	16,762	(34)	<0.001
2 to <3 cm	16,704	(18)	2358	(14)	5491	(33)	8494	(51)	
3 to <4 cm	6728	(7)	858	(13)	1327	(20)	4336	(64)	
4+ cm	6149	(7)	618	(10)	809	(13)	4363	(71)	
Unknown	14,774	(16)	2461	(17)	7357	(50)	4676	(32)	
Tumor grade^8^									
Grade 1	20,582	(22)	3316	(16)	10,508	(51)	6376	(31)	<0.001
Grade 2	37,751	(40)	5124	(14)	16,638	(44)	15,366	(41)	
Grade 3	22,014	(24)	2731	(12)	8176	(37)	10,720	(49)	
Grade 4	1356	(1)	158	(12)	454	(33)	714	(53)	
Grade 9	11,893	(13)	1815	(15)	3915	(33)	5455	(46)	
Number of positive lymph nodes									
0	55,514	(59)	4023	(7)	26,585	(48)	24,304	(44)	<0.001
1	8387	(9)	591	(7)	3310	(39)	4392	(52)	
2–5	7349	(8)	441	(6)	2122	(29)	4686	(64)	
6+	3078	(3)	169	(5)	576	(19)	2254	(73)	
Unknown	19,268	(21)	7920	(41)	7098	(37)	2995	(16)	
Tumor markers									
Other	29,968	(32)	5234	(17)	12,131	(40)	11,533	(38)	<0.001
Positive	54,103	(58)	6849	(13)	23,964	(44)	22,387	(41)	
Negative	9525	(10)	1061	(11)	3596	(38)	4711	(49)	
Number of stable^3^ comorbidities									
0	15,770	(17)	1774	(11)	6464	(41)	6806	(43)	<0.001
1	19,141	(20)	2300	(12)	8413	(44)	7993	(42)	
2	18,634	(20)	2481	(13)	8180	(44)	7634	(41)	
3	40,051	(43)	6589	(16)	16,634	(42)	16,198	(40)	
Number of unstable^4^ comorbidities									
0	75,360	(81)	9755	(13)	33,127	(44)	30,793	(41)	<0.001
1	13,875	(15)	2451	(18)	5222	(38)	5894	(42)	
2	3189	(3)	671	(21)	1027	(32)	1396	(44)	
3+	1172	(1)	267	(23)	315	(27)	548	(47)	

Breast cancer stage of diagnosis was classified on TNM staging system (tumor, node, and metastasis) which is based on the criteria of the American Joint Commission on Cancer. Localized disease includes stages 1, 2a, and 2b; late/advanced stage is defined as stage ≥2b. Tumor markers: positive means estrogen receptor positive; negative means estrogen receptor negative. *N* = 93,596; (*n* **= **2130 women did not receive any treatment, received only other (e.g., hormonal) treatment, or were missing treatment information. BST, breast conserving surgery; RT, radiation therapy; MST; mastectomy.

1 Asian Pacific Islander category includes Chinese, Japanese, Filipino, and Hawaiian women.

2 Other/Unknown and Native American categories were combined due to small numbers.

3 Stable comorbidities were defined as having potential to affect daily activity. Examples include arthritis, osteoporosis, depression, diabetes, thyroid disorders, stable coronary artery disease, and peptic ulcer disease.

4 Unstable comorbidities were defined as life threatening or difficult to control with less than 5 year predicted mortality. Examples include severe heart failure, end-stage pulmonary disease, end-stage liver disease, renal disease, and diabetes with complications.

5 BCS (breast conserving surgery) was classified as suboptimal therapy.

6 BCS + RT (breast conserving surgery and radiation treatment) was classified as preferred surgical therapy.

7 MST ± RT (mastectomy with or without radiation therapy) was classified as other surgery; definitive surgical therapy was defined as BCS + RT or MST ± RT.

8 Tumor grade was classified as well differentiated (grade 1), moderately differentiated (grade 2), poorly differentiated (grade 3), anaplastic (grade 4), or Unknown (grade 9).

The linked SEER-Medicare claims records identified patients with breast cancer surgeries with complete tumor resection performed within 6 months of primary diagnosis. Medicare procedure and revenue center codes were used to identify RT claims 12–19.

Information on sociodemographic characteristics including age and race/ethnicity was ascertained from the linked SEER-Medicare database. Socioeconomic status was estimated by area-based socioeconomic measures taken from patients' residential zip codes including median household income, percentage of residents living below the poverty level, and percentage of residents aged 25 years or older who never completed high school 16–17. Tumors were categorized by size, lymph node involvement, and hormone receptor status 14.

Information on comorbid conditions 11, classified by organ system and severity, was collected using ICD-9-CM codes to identify 38 comorbid conditions as previously described 18,19. The comorbidity measures incorporated more recent versions of ICD-9-CM and were used to identify “stable” and “unstable” comorbidities based on clinical seriousness as reflected in 5-year mortality risk. We defined comorbidities that are life threatening or difficult to control, such as congestive heart failure, cardiac arrhythmias, and end-stage liver disease as “unstable,” and less serious conditions, such as arthritis, depression, and diabetes, with potential to influence daily activity as “stable” 3.

Inpatient, outpatient, and physician–supplier claims were reviewed for 2 years before the breast cancer diagnosis to determine the prevalence of comorbidities during that period, excluding the month of cancer diagnosis. We used a rule-out algorithm to improve agreement of diagnostic information from medical records and Medicare claims 18,20. To estimate total comorbidity burden, we used the number of stable comorbidities. Unstable comorbidities were collapsed into a single group as the prevalence of multiple unstable conditions was very low.

Women were classified as having RT if they had >2 claims for radiation within 9 months from surgery 3–6. Surgical treatment was characterized as breast conservation surgery only (BCS), BCS with radiation (BCS + RT), and MST with or without RT (MST ± RT). The last was considered as one group as few women received postmastectomy RT.

This study was approved for exemption by the Institutional Review Boards of the University of California, Davis and the California Cancer Registry.

### Statistical analysis

Unadjusted percentages of patients with stage I and II breast cancers who received surgery (by category) and RT were calculated, stratified by year of diagnosis, age, sociodemographic and clinical factors, and tumor characteristics. To estimate total comorbidity burden, stable and unstable comorbidities 11 were considered as absent or present, in which case the number of stable comorbidities was grouped as one, two, and three or more (Table 1).

Definitive surgical therapy was defined as BCS plus RT (BCS + RT) or MST with or without radiotherapy (MST ± RT). Suboptimal surgical therapy was defined as BCS without RT (BCS). BCS + RT was defined as preferred surgical therapy.

The *χ*^2^ test was used to test for differences in categorical variables and the Student *t*-test for differences in continuous variables. In age-adjusted analysis, use of BCS + RT was compared with BCS alone, and use of MST ± RT was compared with BCS + RT, among women with and without stable or unstable comorbidities, with further stratification by race/ethnicity.

In age-adjusted analyses, the use of definitive surgical therapy and preferred surgical therapy was compared to suboptimal surgical therapy, stratified by race/ethnicity and comorbidity burden. We constructed separate logistic regression models to estimate the odds of a woman receiving preferred surgical therapy (BCS + RT) (model 1) or definitive surgical therapy (BCS + RT and MST ± RT) (model 2), compared with suboptimal surgical therapy (BCS alone), based on age, race/ethnicity, and comorbidity burden, adjusted for sociodemographic factors, SEER area, and tumor characteristics. The method of recycled probabilities was used to generate adjusted probability estimates for different surgical treatments among patients with specific combinations of age, race/ethnicity, and comorbidity burden.

All analyses were performed with SAS software, version 9.2 (SAS Institute, Inc., Cary, NC).

## Results

**Figure 1:**
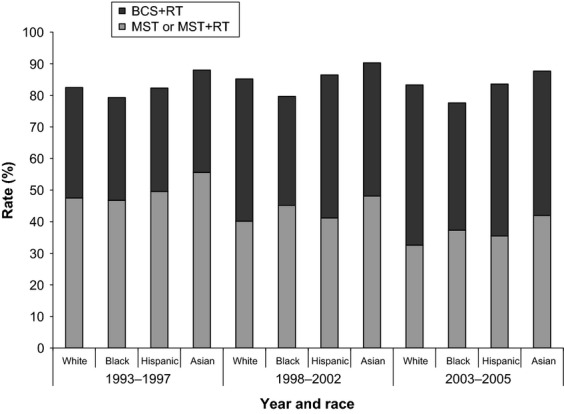
Percentage of women in linked Surveillance, Epidemiology, and End Results (SEER)-Medicare data with early-stage breast cancer who received mastectomy with or without radiation therapy (RT), and breast conserving surgery with RT (BCS ± RT), stratified by race/ethnicity over time, 1993–2005.

Unadjusted use of BCS, BCS + RT, and MST with or without RT, among Medicare beneficiaries diagnosed with stage I or II breast cancer in SEER areas between 1993 and 2005, is shown in Table 1, Over this period, MST use decreased by 15% (48% to 33%) while BCS + RT use increased by 15% (35% to 50%). Preferred surgical therapy (BCS + RT) was also significantly associated with age (from 52% at 67–69 years to 9% at ≥90 years), race/ethnicity (43% of White vs. 36% of Black women), area of residence (45% of large metropolitan vs. 27% of rural residents), neighborhood poverty (35% of residents of lower income neighborhoods vs. 45% of residents of higher income neighborhoods), marital status (49% of married vs. 38% of unmarried women), AJCC stage (51% of stage I vs. 30% of stage II), tumor size (50% at <2 cm vs. 13% at ≥4 cm), tumor grade (51% of well differentiated vs. 33% of anaplastic), number of positive lymph nodes (48% at zero vs. 19% at six or more), and unstable comorbidities (44% with zero vs. 27% with three or more). MST use was significantly associated with residence in a rural or low-income area, stage II disease, tumor size and grade ≥3, positive lymph nodes, and negative estrogen receptors. Among women with early-stage breast cancer, MST use decreased by about 15% among White, Hispanic, and Asian women, and by about 10% among Black women, between 1993–1997 and 2003–2005 (Fig. 1).

A total of 48% of black women with ≥3 stable comorbidities received BCS without radiotherapy, compared with 44% of White, 45% of Hispanic, and 40% of Asian women (data not shown). As displayed in Table 2, age-adjusted analyses showed that women with unstable comorbidities had consistently and significantly higher odds of receiving BCS without RT versus BCS + RT, and receiving MST (with or without RT) versus BCS + RT, across all racial/ethnic groups. Among white and Asian women, but not among Black and Hispanic women, a higher burden of stable comorbidities was associated with reduced MST use.

**Table:** Age-adjusted odds ratios and 95% confidence Intervals of suboptimal surgical treatment (BCS alone vs. BCS + RT) and mastectomy (vs. BCS + RT) among female medicare beneficiaries diagnosed with early-stage breast cancer from 1993 to 2005, overall and stratified by race/ethnicity and comorbidity (*N* = 93,596)

	BCS^3^ versus BCS + RT^4^ (*N* = 42,192)				MST ± RT^5^ versus BCS + RT^4^ (*N* = 65,233)					
Stratum	Level	*N* (%)	OR (95% CI)	*N* (%)	OR (95% CI)
All women					
Overall comorbidity	No	7650 (14)	reference	14,202 (16)	reference
	Yes	45,185 (86)	0.99 (0.92–1.05)	65,920 (84)	**0.84 (0.1–0.87)**
Number of stable comorbidities^1^	0	8238 (16)	reference	13,270 (17)	reference
	1	10,713 (20)	**0.88 (0.81–0.94)**	16,406 (21)	**0.87 (0.83–0.91)**
	2	10,661 (20)	**0.87 (0.80–0.93)**	15,814 (20)	**0.82 (0.79–0.86)**
	3+	23,223 (44)	1.02 (0.95–1.09)	32,832 (41)	**0.82 (0.79–0.86)**
Unstable comorbidity^2^	No	42,882 (81)	reference	63,920 (82)	Reference
	Yes	9953 (19)	**1.52 (1.45–1.61)**	14,402 (18)	**1.22 (1.18–1.27)**
White					
Overall comorbidity	No	6674 (14)	reference	10,760 (16)	Reference
	Yes	39,556 (86)	0.97 (0.91–1.04)	57,285 (84)	**0.83 (0.8–0.86)**
	*P*-Wald Chi-Square <0.001^1^				*P*-Wald Chi-Square <0.001					
Number of stable comorbidities^1^	0	7161 (15)	reference	11,467 (17)	Reference
	1	9486 (21)	**0.87 (0.80–0.95)**	14,407 (21)	**0.86 (0.82–0.90)**
	2	9329 (20)	**0.84 (0.78–0.91)**	13,828 (20)	**0.82 (0.78–0.87)**
	3+	20,254 (44)	1.01 (0.94–1.08)	28,343 (42)	**0.81 (0.78– 0.85)**
Unstable comorbidity^2^	No	37,787 (82)	reference	56,007 (82)	Reference
	Yes	8443 (18)	**1.53 (1.45–1.62)**	12,038 (18)	**1.20 (1.16–1.25)**
Black					
Overall comorbidity	No	300 (11)	reference	482 (12)	reference
	Yes	2394 (89)	1.19 (0.89–1.58)	3532 (88)	1.0 (0.83–1.23)
	*P*-Wald Chi-Square = 0.389				*P*-Wald Chi-Square = 0.067					
Number of stable comorbidities^1^	0	354 (13)	reference	555 (14)	reference
	1	464 (17)	1.11 (0.81–1.53)	730 (18)	1.07 (0.85–1.33)
	2	579 (21)	1.1 (0.81–1.50)	808 (20)	0.87 (0.7–1.08)
	3+	1297 (48)	1.24 (0.94–1.64)	1921 (48)	1.08 (0.89–1.31)
Unstable comorbidity^2^	No	1899 (70)	reference	2829 (70)	reference
	Yes	795 (30)	**1.23 (1.02–1.48)**	1185 (30)	**1.18 (1.03–1.35)**
Hispanic					
Overall comorbidity	No	364 (18)	reference	578 (18)	reference
	Yes	1716 (83)	0.86 (0.65–1.15)	2627 (82)	0.88 (0.74–1.06)
	*P*-Wald Chi-Square = 0.314				*P*-Wald Chi-Square = 0.202					
Number of stable comorbidities^1^	0	385 (19)	reference	618 (19)	reference
	1	374 (18)	0.71 (0.49–1.02)	597 (19)	0.86 (0.69–1.08)
	2	387 (19)	0.87 (0.61–1.23)	571 (18)	0.81 (0.64–1.02)
	3+	934 (45)	0.82 (0.61–1.10)	1419 (44)	**0.82 (0.67–0.99)**
Unstable comorbidity^2^	No	1665 (80)	reference	2523 (79)	reference
	Yes	415 (20)	**1.34 (1.03–1.73)**	682 (21)	**1.38 (1.16–1.63)**
Asian					
Overall comorbidity	No	251 (17)		505 (19)	
	Yes	1257 (83)	0.88 (0.61–1.26)	2178 (81)	**0.74 (0.61–0.91)**
	*P*-Wald Chi-Square = 0.532				*P*-Wald Chi-Square = 0.003					
Number of stable comorbidities^1^	0	272 (18)	reference	547 (20)	reference
	1	329 (22)	0.77 (0.50–1.18)	599 (22)	**0.78 (0.61–0.98**)
	2	297 (20)	0.95 (0.62–1.45)	527 (20)	0.80 (0.63– 1.02)
	3+	610 (40)	0.81 (0.55–1.18)	1010 (38)	**0.67 (0.54– 0.82)**
Unstable comorbidity^2^	No	1261 (84)	reference	2251 (84)	Reference
	Yes	247 (16)	**1.49 (1.06–2.08)**	432 (16)	**1.12 (0.9–1.38)**

Statistically significant effects are shown in bold. BST, breast conserving surgery; RT, radiation therapy; MST; mastectomy; OR, odds ratio; CI, confidence interval.

1 *P*-values for Wald Chi-Square tests (Type 3 analysis of effects) for categorical variables with more than two categories.

1 Stable comorbidities were defined as having potential to affect daily activity. Examples include arthritis, osteoporosis, depression, diabetes, thyroid disorders, stable coronary artery disease, and peptic ulcer disease.

2 Unstable comorbidities were defined as life threatening or difficult to control with >5 year predicted mortality. Examples include severe heart failure, end-stage pulmonary disease, end-stage liver disease, renal disease, and diabetes with complications.

3 BCS (breast conserving surgery) was classified as suboptimal therapy.

3 BCS + RT (breast conserving surgery and radiation treatment) was classified as preferred surgical therapy.

5 MST ± RT (mastectomy with or without radiation therapy) was classified as other surgery; definitive surgical therapy was defined as BCS + RT or MST ± RT.

Table 3 displays the results of multivariate regression models examining significant independent predictors for receiving BCS alone (vs. BCS + RT or any definitive surgical therapy), and for receiving MST with or without RT (MST ± RT vs. BCS + RT). Older age (≥75 years) was associated with omission of RT after breast conservation and with higher MST use. Black women had higher odds of receiving BCS alone (compared with either BCS + RT or any definitive surgical therapy) than White women (odds ratio 2 1.28, 95% confidence interval 3: 1.14–1.45 and OR 1.33, 95% CI: 1.20–1.47, respectively). Receipt of BCS alone (compared with any definitive surgical therapy) was also independently associated with neighborhood socioeconomic status, unmarried status (OR 1.18, 95% CI: 1.12–1.23), tumor size (OR 0.78, 95% CI: 0.69–0.87 for tumors ≥4 cm vs. <2 cm), tumor grade (OR = 0.89, 0.88, and 0.81 for grades 2–4 vs. 1, respectively; see Table 3 for CIs), stable comorbidities (OR = 0.76, 0.71, and 0.72 for 1, 2, and 3 vs. 0 stable comorbidities, respectively; see Table 3 for CIs), and unstable comorbidities (OR 1.20, 95% CI: 1.14–1.28). Interactions between stable and unstable comorbidities and demographic characteristics (age, race/ethnicity categories) were not statistically significant.

**Table:** Multivariable regression models for suboptimal surgical treatment (BCS alone vs. BCS + RT; BCS alone vs. BCS + RT or mastectomy) and mastectomy (vs. BCS + RT) among female medicare beneficiaries diagnosed with early-stage breast cancer from 1993 to 2005 (*n* = 93,596)

Patient or tumor characteristic	BCS^1^ versus BCS + RT^2^ (*n* = 42,192) Model 1 Fit Statistics: Generalized R^2^ = 0.36, C^4^ = 0.82	BCS^1^ versus BCS + RT^2^ and MST ± RT^3^ (*n* = 77,476) Model 2 Fit Statistics: Generalized R^2^ = 0.31, C = 0.81	MST ± RT^3^ versus BCS + RT^2^ (*n* = 65,233) Model 3 Fit Statistics: Generalized R^2^ = 0.26, C = 0.75
	Adjusted OR (95% CI)	Adjusted OR (95% CI)	Adjusted OR (95% CI)
Age in years			
70–74 versus 67–69	1.02 (0.93–1.12)	0.96 (0.89–1.04)	**1.11 (1.06–1.17)**
75–79 versus 67–69	**1.37 (1.25–1.52)**	**1.12 (1.04–1.22)**	**1.31 (1.24–1.38)**
80–84 versus 67–69	**2.44 (2.22–2.70)**	**1.64 (1.49–1.75)**	**1.92 (1.80–2.04)**
85–89 versus 67–69	**5.56 (5.00–6.25)**	**2.78 (2.50–3.03)**	**3.20 (2.93–3.49)^5^**
≥90 versus 67–69	**14.29 12.50–16.67)**	**4.00 (3.57–4.55)**	**6.90 (5.81–8.18)^5^**
Race/ethnicity			
Asian versus White	1.02 (0.83–1.23)	**0.83 (0.70–0.97)**	**1.85 (1.65–2.07)**
Black versus White	**1.28 (1.14–1.45)**	**1.33 (1.20–1.47)**	**0.98 (0.90–1.07)^5^**
Hispanic versus White	0.93 (0.81–1.09)	0.95 (0.84–1.06)	1.02 (0.94–1.12)
Other versus White	**1.69 (1.23–2.33)**	**1.61 (1.25–2.08)**	1.10 (0.86–1.42)
Socioeconomic status			
Patients living in a census tract where 25% of residents do not have a high school diploma	**0.83 (0.77–0.88)**	**0.92 (0.87–0.97)**	**0.83 (0.80–0.87)**
SEER area of residence			
Big metro versus less urban/rural	**0.69 (0.61–0.80)**	1.10 (0.98–1.22)	**0.59 (0.54–0.64)**
Metro versus less urban/rural	**0.62 (0.55–0.71)**	0.96 (0.87–1.06)	**0.64 (0.59–0.69)**
Urban versus less urban/rural	**0.71 (0.61–0.83)**	0.88 (0.78–1.01)	**0.66 (0.60–0.73)^5^**
Marital status			
Unmarried versus married	**1.33 (1.27–1.43)**	**1.18 (1.12–1.23)**	**1.23 (1.19–1.28)**
Unknown versus married	**1.75 (1.52–2.00)**	**1.61 (1.43–1.82)**	**1.12 (1.00–1.25)**
SEER region of residence			
Connecticut versus Seattle	0.91 (0.80–1.04)	1.03 (0.92–1.15)	**0.64 (0.58–0.69)**
Detroit versus Seattle	1.12 (0.99–1.30)	1.09 (0.97–1.22)	1.07 (0.98–1.16)
Greater California versus Seattle	1.04 (0.93–1.18)	**1.69 (1.01–1.54)**	0.98 (0.91–1.05)
Hawaii versus Seattle	1.18 (0.89–1.56)	1.15 (0.90–1.47)	**0.74 (0.63–0.86)**
Iowa versus Seattle	**1.47 (1.25–1.72)**	1.12 (0.98–1.28)	**1.89 (1.72–2.08)**
Kentucky versus Seattle	1.16 (0.95–1.41)	1.08 (0.92–1.27)	**1.23 (1.09–1.38)**
Los Angeles versus Seattle	1.12 (0.98–1.27)	**1.19 (1.05–1.33)**	**0.70 (0.64–0.76)**
Louisiana versus Seattle	**1.49 (1.23–1.82)**	**1.27 (1.09–1.49)**	**1.46 (1.30–1.64)**
New Jersey versus Seattle	1.11 (0.96–1.28)	**1.39 (1.22–1.56)**	**0.52 (0.47–0.57)**
New Mexico versus Seattle	1.20 (0.99–1.47)	1.14 (0.96–1.35)	1.12 (0.99–1.27)
San Francisco versus Seattle	**1.23 (1.06–1.43)**	**1.20 (1.05–1.37)**	**0.77 (0.70–0.85)**
San Jose versus Seattle	1.11 (0.92–1.33)	0.98 (0.83–1.15)	**1.16 (1.03–1.30)**
Utah versus Seattle	**1.72 (1.43–2.08)**	**1.25 (1.06–1.47)**	**1.31 (1.16–1.46)**
AJCC stage			
Stage 2 versus 1	1.02 (0.91–1.14)	0.99 (0.90–1.09)	**1.19 (1.11–1.27)^5^**
Tumor size			
2 to <3 cm versus 0 to <2 cm	**1.35 (1.23–1.49)**	1.05 (0.97–1.14)	**1.75 (1.65–1.85)**
3 to <4 cm versus 0 to <2 cm	**1.92 (1.67–2.22)**	0.94 (0.84–1.05)	**3.33 (3.05–3.62)**
4+ cm versus 0 to <2 cm	**2.08 (1.75–2.44)**	**0.78 (0.69–0.87)**	**5.79 (5.26–6.38)**
Tumor grade			
Grade 2 versus 1	0.95 (0.88–1.02)	**0.89 (0.84–0.94)**	**1.25 (1.20–1.31)**
Grade 3 versus 1	0.95 (0.88–1.03)	**0.88 (0.81–0.93)**	**1.40 (1.33–1.48)**
Grade 4 versus 1	1.01 (0.79–1.28)	**0.81 (0.66–0.98)**	**1.52 (1.31–1.76)**
Grade unknown versus 1	**1.18 (1.08–1.28)**	**1.10 (1.02–1.19)**	**1.76 (1.66–1.87)**
Number of positive lymph nodes			
1 versus none	1.09 (0.94–1.25)	1.01 (0.90–1.12)	1.04 (0.97–1.12)
2–5 versus none	**1.20 (1.03–1.39)**	0.90 (0.80–1.01)	1.47 (1.36–1.59)
6+ versus none	**1.79 (1.47–2.22)**	1.03 (0.89–1.20)	**2.06 (1.85–2.30)**
Unknown versus none	**5.00 (4.55–5.26)**	**8.33 (7.69–8.33)**	**0.28 (0.27–0.30)**
Tumor marker			
Other versus negative	**1.64 (1.49–1.82)**	**1.45 (1.33–1.59)**	**1.47 (1.38–1.57)**
Positive versus negative	**0.88 (0.81–0.97)**	0.93 (0.87–1.01)	**0.92 (0.87–0.98)**
Stable comorbidities			
1 comorbidity versus 0	**0.90 (0.83–0.99)**	**0.76 (0.71–0.82)**	**0.91 (0.86–0.96)**
2 comorbidities versus 0	**0.86 (0.79–0.94)**	**0.71 (0.66–0.76)**	**0.90 (0.85–0.95)**
3 comorbidities versus 0	1.00 (0.89–1.04)	**0.72 (0.68–0.78)**	**0.92 (0.87–0.97)**
Unstable comorbidities			
Yes versus No	**1.35 (1.27–1.43)**	**1.20 (1.14–1.28)**	**1.27 (1.21–1.33)**

Statistically significant effects are shown in bold.

Model 1 compares suboptimal therapy with preferred surgical therapy among women who received breast conserving surgery (BCS vs. BCS + RT). Model 2 compares suboptimal therapy with definitive surgical therapy among women who received any primary surgery (BCS vs. BCS + RT and MST + RT). Model 3 compares mastectomy to BCS + RT among women who received definitive surgical therapy (MST + RT vs. BCS + RT). BST, breast conserving surgery; RT, radiation therapy; MST; mastectomy; OR, odds ratio; CI, confidence interval.

1 BCS (breast conserving surgery) was classified as suboptimal therapy.

2 BCS + RT (breast conserving surgery and radiation treatment) was classified as preferred surgical therapy.

3 MST ± RT (mastectomy with or without radiation therapy) was classified as other surgery; definitive surgical therapy was defined as BCS + RT or MST ± RT.

4 Estimated area under receiver operation characteristic (ROC) curve.

5 *P*-values for Wald Chi-Square tests (Type3 analysis of effects) for all other categorical variables with more than two categories were <0.005.

Significant independent predictors of MST with or without RT (vs. BCS + RT) were age older than ≥75, Asian (vs. White) race/ethnicity (OR 1.85, 95% CI: 1.65–2.07), neighborhood socioeconomic status, area of residence, marital status (unmarried vs. married) (OR 1.23, 95% CI: 1.19–1.28), stage II versus stage I (OR 1.19, 95% CI: 1.11–1.27), tumor size (OR 1.75, 3.33, and 5.79 for 2 to <3 cm, 3 to <4 cm, and ≥4 cm vs. <2 cm, respectively; see Table 3 for CIs), tumor grade (OR 1.25, 1.40, and 1.52 for grades 2, 3, and 4 vs. 1, respectively; see Table 3 for CIs), positive lymph nodes (OR 1.47, 95% CI: 1.36–1.59 for 2–5 vs. 0 nodes; OR = 2.06, 95% CI: 1.85–2.30 for ≥6 vs. 0 nodes), stable comorbidities (OR = 0.91, 0.90, and 0.92 for 1, 2, and 3 vs. 0 stable comorbidities, respectively; see Table 3 for CIs), and unstable comorbidities (OR 1.27, 95% CI: 1.21–1.33). Use of both BCS alone and MST varied across SEER areas of residence (Table 3). Interactions between stable and unstable comorbidities and demographic characteristics (age, race/ethnicity categories) were not statistically significant.

Table 4 displays the predicted probability of receiving each type of treatment, stratified by age, race/ethnicity, and stable and unstable comorbidity, and adjusted for all of the other factors in the Table 3 models. In absolute terms, Black women were 4–5% more likely to receive suboptimal therapy with BCS alone, even after adjusting for all available patient, tumor, and regional characteristics. Similarly, women with unstable comorbidities were more likely to receive suboptimal therapy than women without unstable comorbidities (17.3% vs. 15.4% among those who received any surgical treatment for local stage cancer), even though women with unstable comorbidities were also about 5% more likely to receive MST. Conversely, stable comorbidities were associated with a lower likelihood of suboptimal therapy and a lower likelihood of MST. Asian women had a 12% higher likelihood of receiving MST with or without RT, relative to other racial/ethnic groups.

**Table:** Predicted probabilities of receiving suboptimal surgical treatment (BCS alone vs. BCS + RT; BCS alone vs. BCS + RT or Mastectomy) and mastectomy (vs. BCS + RT) among female medicare beneficiaries diagnosed with early-stage breast cancer from 1993 to 2005, using fitted models in Table 3 and recycled prediction method (*N* = 93,596)

Patient or tumor characteristic	Model 1	Model 2	Model 3
	Predicted probability of receiving BCS^1^ versus BCS + RT^2^	Predicted probability of receiving BCS^1^ versus BCS + RT^2^ or MST ± RT^3^	Predicted probability of receiving MST ± RT^3^ versus BCS + RT^2^
Age in years			
67–69	0.168	0.123	0.450
70–74	0.168	0.120	0.471
75–79	0.206	0.135	0.505
80–84	0.298	0.174	0.583
85–89	0.455	0.244	0.682
≥90	0.649	0.306	0.806
Race/Ethnicity			
White	0.248	0.139	0.508
Black	0.281	0.188	0.504
Hispanic	0.236	0.151	0.512
Asian	0.318	0.211	0.630
Other	0.245	0.157	0.527
Stable comorbidities			
0	0.260	0.186	0.527
1	0.243	0.157	0.508
2	0.236	0.150	0.507
3+	0.250	0.152	0.511
Unstable comorbidities			
No	0.239	0.154	0.504
Yes	0.278	0.173	0.552

BST, breast conserving surgery; RT, radiation therapy; MST; mastectomy.

1 BCS (breast conserving surgery) was classified as suboptimal therapy.

2 BCS + RT (breast conserving surgery and radiation treatment) was classified as preferred surgical therapy.

3 MST ± RT (mastectomy with or without radiation therapy) was classified as other surgery; definitive surgical therapy was defined as BCS + RT or MST ± RT.

## Discussion

Among elderly female Medicare beneficiaries, comorbidities were independently associated with surgical treatment. Specifically, stable comorbidities were associated with less use of suboptimal surgical treatment (BCS alone) and less use of MST instead of BCS + RT, whereas unstable comorbidities were associated with more use of suboptimal surgical treatment and more use of MST instead of BCS + RT. We found that age effects on treatment patterns were independent of comorbidity status and older age was associated strongly with omission of radiation after breast conservation, and MST instead of BCS + RT. The probability of receiving treatment with BCS alone or MST increased with age, from 12.3% of women aged 67–69 years receiving any surgery, and 45.0% of women aged ≥90 years receiving definitive surgical treatment to 30.6% and 80.6%, respectively, among women aged ≥90 years. These findings are consistent with previous studies showing that chronologic age was associated with suboptimal therapy independent of comorbidities, suggesting that physicians may be undertreating healthy older women 4.

The race and comorbidity stratified data showed that the choice of treatment (BCS alone vs. BCS + RT and MST vs. BCS + RT) was associated with unstable comorbidities across all race/ethnic groups. Although definitive local therapy rates (BCS + RT or MST) are improving over time in minority patients with breast cancer, Black women still show a lag compared with women in other racial/ethnic categories (Fig. 1). Even after adjusting for all other patient, tumor, and regional characteristics, Black women had 4–5% higher probability of receiving treatment with BCS alone, and no higher probability of receiving MST, compared with White women. The finding that Black women were more likely than women in other racial/ethnic categories to receive suboptimal initial treatment with BCS alone is consistent with previous reports 8,11. Asian race/ethnicity was predictive of receiving MST with or without radiation treatment; these findings are also in agreement with previous studies 3. Use of MST instead of BCS + RT among Asian women may be due to smaller breasts and inability to preserve adequate breast tissue with BCS due to higher tumor-to-breast size ratios compared with women in other racial/ethnic categories 3.

In agreement with a prior report 23, unmarried women were also less likely to receive preferred treatment with BCS + RT. As marital status may be a proxy for social support, treatment choices by unmarried women may reflect a lack of social support. Similarly, metropolitan residence and SEER area of residence may be important proxies for geographic and cultural access to appropriate cancer care 24.

The comorbidity classification used in these analyses incorporates judgments regarding the severity of illness. Using this classification, we were able to capture greater variance in breast cancer therapy related to comorbidity than in analyses using other comorbidity measures 25–26. Even after adjusting for all measured patient, tumor, and regional characteristics, stable comorbidities were associated with less use of BCS alone among all women who received surgery for early-stage breast cancer (15.0–15.7% vs. 18.6%), and slightly less use of MST among women who received definitive surgical treatment (50.7–51.1% vs. 52.7%), whereas unstable comorbidities were associated with more use of BCS alone (17.3% vs. 15.4%) and more use of MST (55.2% vs. 50.4%). These effects may reflect improved access to services and perhaps stronger physician–patient relationships among women with stable comorbidities. On the other hand, concern about unstable comorbidities may be limiting appropriate use of RT in this clinical setting.

Study strengths include the large sample size, the geographic, racial, and ethnic diversity, and the use of 2 years of prior inpatient and outpatient claims to estimate comorbidity burden and severity. These data cover the largest available population of breast cancer patients with detailed data on cancer diagnosis from U.S. tumor registries, with linked administrative data from Medicare claims to identify comorbid diagnoses and treatment modalities. Results are likely generalizable to elderly women with breast cancer in the United States, as they reflect community based, usual care for the elderly and include racial/ethnic minorities in sufficient numbers to evaluate variations in care across these groups.

Study limitations include the use of administrative claims generated for billing rather than for research purposes. However, treatment misclassification is unlikely, as previous SEER-Medicare data analyses have demonstrated substantial concordance with chart abstraction results 12–20. While direct information about socioeconomic, education, or insurance status was not available, the use of area-level measures as a proxy for individual SES is a standard approach 17,27 and unlikely to introduce substantial bias.

In summary, all comorbidities are not equivalent in terms of how they affect clinical decision making for women with early-stage breast cancer. Stable comorbidities have a modest but favorable impact on increasing use of definitive surgical treatment and decreasing use of MST. Unstable comorbidities modestly but adversely influence receipt of definitive and preferred early-stage breast cancer therapy. Black race/ethnicity was associated with higher probability of receiving suboptimal treatment, independent of comorbidities, although we do not know whether this effect was due to clinicians' failure to offer RT or patients' failure to accept it. In either case, racial/ethnic and regional disparities in surgical treatment for early-stage breast cancer persist, and are not explained by comorbid illness or any other reported patient or tumor characteristics. These disparities should remain an important focus of attention for those involved in the organization and delivery of care to women with breast cancer in the U.S.

## Conflict of Interest

None declared.
